# A general derivation and quantification of the third law of thermodynamics

**DOI:** 10.1038/ncomms14538

**Published:** 2017-03-14

**Authors:** Lluís Masanes, Jonathan Oppenheim

**Affiliations:** 1 Department of Physics & Astronomy, University College of London, London WC1E 6BT, UK

## Abstract

The most accepted version of the third law of thermodynamics, the unattainability principle, states that any process cannot reach absolute zero temperature in a finite number of steps and within a finite time. Here, we provide a derivation of the principle that applies to arbitrary cooling processes, even those exploiting the laws of quantum mechanics or involving an infinite-dimensional reservoir. We quantify the resources needed to cool a system to any temperature, and translate these resources into the minimal time or number of steps, by considering the notion of a thermal machine that obeys similar restrictions to universal computers. We generally find that the obtainable temperature can scale as an inverse power of the cooling time. Our results also clarify the connection between two versions of the third law (the unattainability principle and the heat theorem), and place ultimate bounds on the speed at which information can be erased.

## Abstract (web summary)

The third law of thermodynamics, first formulated in 1912, states that any process cannot reach absolute zero temperature in finite time. Here, the authors derive the third law in the quantum regime as a bound on the resources necessary to cool a system to any temperature.

The third law of thermodynamics has a controversial past and a number of formulations due to Planck, Einstein and Nernst. Walther Nernst's first formulation of the third law of thermodynamics^1^, now called the heat theorem, was the subject of intense discussion^2^. Nernst claimed that he could prove his heat theorem using thermodynamical arguments while Einstein, who refuted several versions of Nernst's attempted derivation, was convinced that classical thermodynamics was not sufficient for a proof, and that quantum theory had to be taken into account. Max Planck's formulation^3^: when the temperature of a pure substance approaches absolute zero, its entropy approaches zero; may hold for many crystalline substances, but it is not true in general, and formulations due to Einstein^4^ and Nernst^5^ were for some time considered to be typically true from an experimental point of view, but sometimes violated.

A modern understanding of entropy and quantum theory takes the heat theorem outside the realm of thermodynamics. Nernst's version states that: at zero temperature, a finite size system has an entropy *S*, which is independent of any external parameters *x*, that is *S*(*T*, *x*_1_)−*S*(*T*, *x*_2_)→0 as the temperature *T*→0. Since we now understand the entropy at zero temperature to be the logarithm of the ground-state degeneracy, the validity of the heat theorem is contingent on whether the degeneracy changes for different parameters of the Hamiltonian. One can easily find families of Hamiltonians, which satisfy or violate the heat theorem^6^,^7^. Here however, we concern ourselves with the question of Nernst's unattainability principle^8^, that Nernst introduced to support his attempted derivations of his heat theorem and counter Einstein's objections. We can understand it as saying that putting a system into its ground state requires infinite time or an infinite number of steps. Nernst argues that if the heat theorem could be violated, then it would be possible to violate the unattainability principle (Fig. 1). We will see that this is not the case. Although one can potentially cool at a faster rate in systems violating the heat theorem, we show that the unattainability principle still holds. The bound we obtain quantifies the extent to which a change in entropy at *T*=0 affects the cooling rate.

Independently of this debate, the validity of the unattainability principle has remained open. Although formulated in 1912, there has been no general proof of the principle, despite the central importance that cooling has in enabling quantum phenomena in optical, atomic and condensed matter systems. Quantum computation, precision measurements, quantum simulations and the manipulation of materials at the atomic scale, all rely on extreme cooling. Currently, we only know that certain cooling protocols, whether it be laser cooling, algorithmic cooling, dynamic cooling or the traditional alternating adaibatic and isothermic reversible operations, require infinite time to cool a system to absolute zero. The analysis of particular cooling protocols^9^,^10^ yields quantitative bounds on how fast cooling can take place, provided one makes certain physical assumptions. While for other protocols and physical assumptions, there are claims of violations of the third law^11^, followed by counter-claims^12^ and counter–counter-claims^13^. The limitation of these results is that, certain physical assumptions may not be valid at arbitrarily low temperature, or that certain protocols may not be optimal. Without a proof based on first principles, the validity of the third law is in question and cannot be held in the same esteem as the other laws of thermodynamics.

A number of recent works analyse a process closely related to cooling to absolute zero: erasing information or generating pure states. In refs 14, 15, 16 it is shown that, regardless of the amount of time invested, these processes are strictly impossible if the reservoir is finite-dimensional. However, strict unattainability in the sense of Nernst is not really a physically meaningful statement. Rather, one wants to obtain a finite bound to how close one can get to the desired state with a finite amount of resources or within a given time. Some interesting steps in this direction are taken in ref. 17, where they obtain a bound in terms of the dimension of the reservoir, but not one that can be translated into time. It also requires the dimension of the reservoir to be finite, an assumption that is not needed to derive our unattainability result here, and something which rarely holds in real setups. In fact, we shall see that the physical mechanism which enforces our third law is not dimension, but the profile of the density of states of the reservoir^18^. On the other hand, argues that for a qubit, one can produce a pure state to arbitrary accuracy as the time invested increases. This, however, requires that the work injected in the bath fluctuates around its mean value by an unbounded amount (this is also necessary in ref. 17). A fact that becomes more relevant when cooling systems much larger than a qubit.

In the present article, we bound the achievable temperature by resources such as the volume of the reservoir and the largest value by which the work can fluctuate. This in itself can be said to constitute a version of the third law. However, we also argue that, in any process implemented in finite time, these two resources must remain finite too. When the scaling of these resources with time has a standard form (explained below), and the heat bath consists of radiation, our third law provides the following relation between the lowest achievable temperature 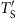 and time *t*


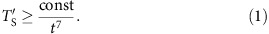


We believe this to be the first derivation of a quantitative lower bound on the temperature in terms of cooling resources, confirming the general validity of the models and conjecture in ref. 9 (although we do not require the system to be continually thermal, we are able to get a bound which is more general than the differential equation postulated there).

The question of how much time a state transformation takes is a very natural question to ask in the field of theoretical computer science, which generally tries to quantify the resources needed to perform a task. In the case of lower bounding the resources to perform a computation, this can be in terms of the number of basic steps or gates (given a certain energy). It is thus no surprise, that the techniques we will use come from recent efforts to construct a theory of thermodynamics based on fundamental principles of quantum information theory^19^,^20^,^21^,^22^,^23^,^24^,^25^,^26^,^27^,^28^,^29^,^30^,^31^,^32^,^33^,^34^,^35^. Traditionally, thermodynamics has been mostly concerned with large, classical systems, but these recent results also apply to microscopic quantum systems in arbitrary non-equilibrium states coupled to a heat reservoir, as is the case here. We thus wish to contribute to the programme of deriving the whole of thermodynamics from more fundamental principles.

## Results

### Physical setup

Our goal is to provide ultimate quantitative bounds applicable to any cooling procedure—namely, we wish to find a lower bound for the temperature that a system can reach after any process which uses some given resources or lasting some given time *t*. Therefore, we must allow for the most general quantum transformation, that is, those that respect total energy conservation and are microscopically reversible (unitary). This general setup includes thermodynamically irreversible protocols and also unrealistic protocols where total control of the microscopic degrees of freedom of the bath is required. Surprisingly, we will find here, as was found for the case of the second law^25^,^27^,^29^,^30^, that having such unrealistic degree of control does not appear to give one an advantage over having very crude control.

We will show that the density of states of the reservoir assisting the cooling process has an important impact on how fast a system can be cooled. (The density of states Ω(*E*) is the number of states with energy *E*.) We see that the faster Ω(*E*) grows, the lower the temperature that can be achieved with fixed resources or in a fixed amount of time. Even more: if Ω(*E*) grows exponentially or faster, then cooling to absolute zero in finite time is in principle possible, allowing for a violation of the third law. However, we will see that exponential or super-exponential Ω(*E*) should be regarded as unphysical. This becomes more intuitive when expressed in terms of the (micro-canonical) heat capacity *C*(*E*), related to *S*(*E*)=ln Ω(*E*) via


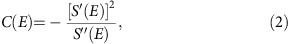


where primes represent differentials. If Ω(*E*) grows exponentially or faster, then *C*(*E*) is infinite or negative, which is regarded as unphysical. If Ω(*E*) is sub-exponential, then *C*(*E*) is positive. And, the faster Ω(*E*) grows, the larger *C*(*E*) is. Only a reservoir with infinite-dimensional Hilbert space can keep *S*(*E*) growing for all *E*. And indeed, infinite-dimensional reservoirs are the ones that allow for faster cooling. However, our results are general and also apply to the finite-dimensional case.

Suppose that we want to cool a quantum system with Hilbert space dimension *d*, and Hamiltonian *H*_S_ having ground-state degeneracy *g*, gap above the ground state Δ and largest energy *J*. What are the resources required to do so?

### Fundamental assumptions

Let us specify the setup more concretely and collect the assumptions we will adopt (those which come from first principles):

(i) We consider the start of the process to be when the system has not yet been put in contact with the work storage system (the weight) nor the reservoir, so that initially, the global state is *ρ*_S_⊗*ρ*_B_⊗*ρ*_W_. While other initial starting scenario may be of interest, its consideration is beyond the scope of the current paper.

(ii) We allow for the most general quantum transformation on system, bath and weight, which is reversible (unitary) and preserves total energy. This might appear restrictive compared with the paradigms that allow arbitrary interaction terms, however this is not the case, since arbitrary interactions can be incorporated into the model as shown in Appendix H of ref. 27 and in ref. 25, simply by allowing the energy of the work system to fluctuate. In many paradigms, this is implicitly enforced by assuming that all missing energy is counted as work. Paradigms which relax this condition are essentially ignoring the energy transferred to other systems, or treat these other systems as classical. Essentially, we impose energy conservation to ensure we properly account for all energy costs associated with the interaction while the various unitaries or interaction terms simply transfer or take energy from the weight to compensate. The cooling process is thus any transformation of the form


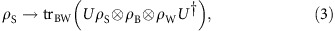


where *U* is a global unitary satisfying


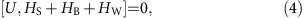


(iii) The work that is consumed within the transformation is taken from the weight. Since we are interested in ultimate limitations, we consider an idealized weight with Hamiltonian having continuous and unbounded spectrum 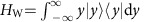. Any other work system can be simulated with this one^30^. We denote by *w*_max_ the worst-case value of the work consumed, that is,


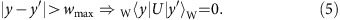


*w*_max_ will generally be much larger than the average work 〈*W*〉. In any physically reasonable process carried out in finite time, one expects it to be finite.

(iv) We also require, as in ref. 29, that the cooling transformation commutes with the translations on the weight. In other words, the functioning of the thermal machine is independent of the origin of energies of the weight, so that it just depends on how much work is delivered from the weight. This can be understood as defining what work is—it is merely the change in energy we can induce on some external system. This also ensures that the weight is only a mechanism for delivering or storing work, and is not, for instance, an entropy dump (see Result 1 in the Supplementary Discussion). It also ensures that the cooling process always leaves the weight in a state that can be used in the next run or the process. Thus


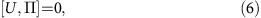


where the Hermitian operator Π acts as 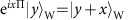 for all 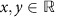. Beyond this, we allow the initial state of the weight *ρ*_W_ to be arbitrary. In particular, it can be coherent, which provides an advantage^27^.

(v) We assume that the bath has volume *V* and is in the thermal state 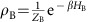 at given inverse temperature 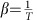, with *Z*_B_ the partition function of the bath. We denote the free energy density of the bath (in the canonical state *ρ*_B_) by 
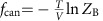
.

(vi) The micro-canonical heat capacity (2) is not negative *C*(*E*) for all energies *E*. This implies that *S*(*E*) is sublinear in *E*. We also prove in the Supplementary Methods that if *S*(*E*) grows linearly or faster, then perfect cooling in finite time is possible.

With these assumptions, we show that to perfectly cool the system to absolute zero, at least one of these two resources, the volume of the bath *V*, or the worst-case value of the work consumed *w*_max_ has to be infinite. Also, we bound the lowest achievable temperature of the system 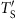 in terms of *V* and *w*_max_.

### Quantifying unattainability from first principles

With assumptions (i)–(vi), we consider two cases, one where the initial and final state are thermal, and one where we allow arbitrary initial and final states. Our first result concerns the former, and states that in any process where the worst-case work injected is *w*_max_, the final temperature of the system cannot be lower than


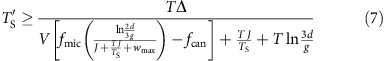


in the large *w*_max_,*V* limit. The micro-canonical free-energy density at inverse temperature *β*_0_ is defined by


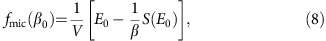


where *E*_0_ is the solution of equation *S*′(*E*_0_)=*β*_0_. Recall that, when the volume of the bath *V* is large, it is usually the case that *f*_mic_(*β*_0_)=*f*_can_(*β*_0_) and these are independent of *V*.

Let us analyse the behaviour of equation (7) in terms of the resources invested. As *w*_max_ grows, *β*_0_ decreases and *f*_mic_ increases, yielding a lower final temperature 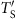. Since all the volume dependence in equation (7) is explicit, hence, a larger *V* also translates into a lower final temperature.

In what follows we provide a bound for the physically relevant family of entropies


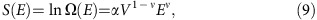


where *α*>0 and *ν*∈[1/2, 1) are two constants. Such an entropy is extensive, and if we set 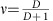 it describes electromagnetic radiation (or any massless bosonic field) in a *D*-dimensional box of volume *V*. It is generally believed that there is no other reservoir that has a density of states growing faster with *E* than this^36^, and certainly none which has *ν*≥1. The later, corresponds to the bath with negative heat capacity discussed earlier, which enables cooling with finite *w*_max_. In the Supplementary Discussion, we adapt bound (7) to the entropy (9), obtaining


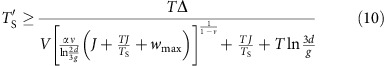


up to leading terms. Now, all the dependence on *V* and *w*_max_ is explicit. In particular, we observe that larger values of *V* and *w*_max_ allow for lower temperatures. And also, larger values of *ν*, which amount to a faster entropy growth, allowing for lower temperatures.

As mentioned above, the cooling processes that we consider are very general. In particular, they can alter the Hamiltonian of the system during the process, as long as the final Hamiltonian is identical to the initial one *H*_S_. This excludes the uninteresting cooling method consisting of re-scaling the Hamiltonian *H*_S_→0. However, our bounds can easily be adapted to process where the final Hamiltonian differs from the initial one, as we will discuss in the conclusion.

Let us now consider the more general case, where neither the initial or final state need be thermal, but can instead be arbitrary. As it is already well known^14^,^15^,^17^,^18^,^30^, the unattainability of absolute zero is not a consequence of the fact that the target state has low energy, but rather that it has low entropy. Hence, this directly translates to the unattainability of any pure state, or more generally, any state with rank *g* lower than the initial state. These type of processes are generally known as information erasure, or purification. Now we analyse the limitations of any processes which takes an arbitrary initial state *ρ*_S_ and transforms it into a final state 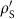 with support onto the *g*-rank projector *P*. We quantify the inaccuracy of the transformation by the error 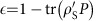. For the sake of clarity, we assume that the system has trivial Hamiltonian *H*_S_=0 (the general case is treated in the Supplementary Discussion), and we denote by *λ*_min_ and *λ*_max_ the smallest and largest eigenvalues of *ρ*_S_. In the Supplementary Methods, we show that any process *ρ*_S_→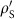 has error


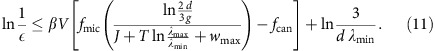


The results presented above, as well as others of more generality presented in the Supplementary Discussion, quantify our ability to cool a system (or more generally, put it into a reduced rank state), in terms of two resources: the volume of the bath *V*, and the worst-case fluctuation of the work consumed *w*_max_. They thus constitute a form of third law, in the sense that they place a bound on cooling, given some finite resources. We now wish to translate this into the time it would take to cool the system, and we will do so, by consider the notion of a thermal machine and making two physically reasonable assumptions.

### Thermal machines

Let us recall that the field of computational complexity is based on the Church-Turing thesis—the idea that we consider a computer to be a Turing machine, and then explore how the time of computation scales with the size of the problem. Different machines may perform differently—the computer head may move faster or slower across the memory tape; information may be stored in bits or in higher dimensional memory units, and the head may write to this memory at different speeds. Nature does not appear to impose a fundamental limit to the dimension of a computer memory unit or the speed at which it may be written. However, for any physically reasonable realization of a computer, and whatever the speed of these operations, it is fixed and finite, and only then do we examine the scaling of time with problem size. And what is important is the overall scaling of the time with input (polynomial or exponential), rather than any constants. Likewise here, we will consider a fixed thermal machine, and we will assume that it can only transfer a finite amount of energy into the heat bath in finite time. Likewise, in a finite time, it cannot explore an infinite size heat bath. A thermal machine which did otherwise would be physically unreasonable.

We can consider both *V* and *w*_max_ as monotonic functions of time *t*. The longer our thermal machine runs, the more work it can pump into the heat bath, and the larger the volume of the bath it can explore. For any particular thermal machine, one can put a finite bound on 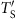 by substituting these functions into equation (10). In particular, if we assume that the interaction is mediated by the dynamics of a local Hamiltonian, then the interaction of a system with a bath of volume *V* and spacial dimension *d* will take time


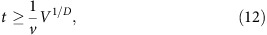


where *v* is proportional to the speed of sound in the bath (or Lieb–Robinson velocity^37^), and *V*^1/*D*^ the linear dimension of the bath. The implementation of general unitaries takes much longer than equation (12), but this serves as a lower bound. Since we are interested here in the scaling of temperature with time, rather than with constant factors, we need not be concerned by the fact that practical thermal machines operate at much slower speeds. Of course, just as with actual computers, thermal machines generally have speeds well below the Lieb–Robinson bound. Note that, despite *V* being finite, the Hilbert space of the bath can be infinite-dimensional. If one wanted to have a bound which was independent of the thermal machine, and independent on the speed of sound which is a property of the bath, then one could always take *v* to be the speed of light. While such a bound would not be practically relevant, it would be fundamental. This is similar to bounds on computation, where to get a fundamental bound, one should take the gate speed to be infinite (since there is no fundamental bound on this) and convert the number of bits used in the process to time by multiplying by the speed of light.

A relationship between worst-case work *w*_max_ and time *t* is obtained by noticing the following. In finite *t* it is not possible to inject into the bath an infinite amount of work. For simplicity, here we assume a linear relationship


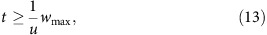


where the constant *u* will depend on the interactions between system and weight. However, we stress that, if a particular physical setup is incorrectly modelled by the relations (12) and (13), then any other bound *t*≥*h*_1_(*w*_max_) and *t*≥*h*_2_(*V*) is also good. As long as *h*_1_ and *h*_2_ are strictly monotonic functions the unattainability principle will hold.

### Limitations using thermal machines

For any particular thermal machine, we can now derive limitations on the temperature that can be reached in a given time *t*. Since the physical system with the fastest entropy growth that we are aware of is radiation, it is worthwhile to dedicate the next paragraph to the case 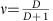 in equation (9), because this should provide a bound with wide validity. Using the particular relations (12) and (13), and substituting them into equation (10), for the case of radiation, we obtain


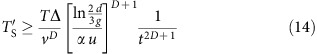


in the large *t* limit. Our bound (14) can be straightforwardly adapted to any other relation *t*≥*h*_1_(*w*_max_) and *t*≥*h*_2_(*V*). It is interesting to observe in equation (14) the relationship between the characteristic time (how long does it takes to cool to a fixed 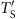) and the size of the system *V*_S_. Exploiting the usual relation ln *d*∝*V*_S_ we obtain the sublinear scaling


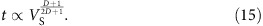


Something concerning about result (11) is that, in the limit *λ*_min_→0 the bound becomes trivial 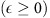. This can be solved by truncating the initial state *ρ*_S_ to the subspace containing the *k* largest eigenvalues and optimizing the resulting bound for 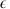 as a function of *k*. Also, this truncation method allows to extend all our results to infinite-dimensional systems (*d*=∞).

## Discussion

We hope the present work puts the third law on a footing more in line with those of the other laws of thermodynamics. These have already been long established, although they've recently been reformulated within the context of other resource theories^19^,^20^,^25^,^27^,^38^,^39^,^40^. Namely, as described in ref. 30, the first law (energy conservation), and unitarity (or microscopic reversibility) describe the class of operations which are allowed within thermodynamics. The zeroth law, is the fact that the only state which one can add to the theory without making it trivial, are the equivalence class of thermal states at temperature *T*. This allows the temperature to emerge naturally. The second law(s), tells us which state transformations are allowed under the class of operations. For macroscopic systems with short-range interactions, there is only one function, the entropy, which tells you whether you can go from one state to another, but in general there are many constraints^25^,^30^,^31^,^32^. The third law quantifies how long it takes to cool a system. We propose to generalize it further: While the second laws tell us which thermodynamical transitions are possible, generalized third laws quantify the time of these transitions. In this context, it would be interesting to explore the time and resource costs of other thermodynamical transitions. It would also be interesting to explore the third law in more restricted physical settings, as well as to other resource theory frameworks, in particular, those discussed in ref. 30.

**Figure 1:**
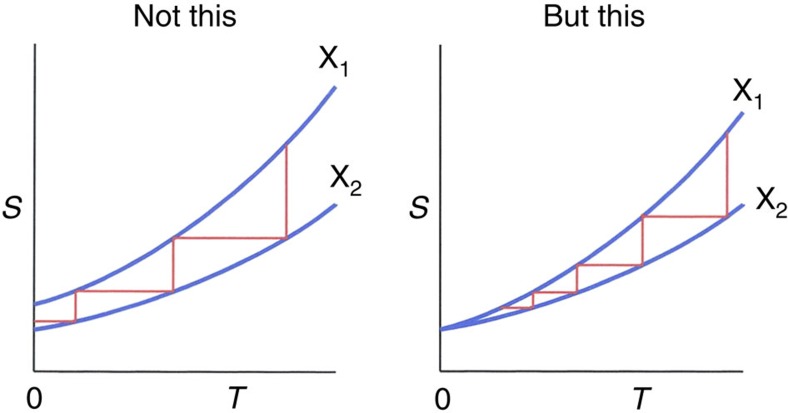
Nernst justification of the unattainability principle. If the heat theorem is violated then perfect cooling can be achieved with a finite number steps. On the right, absolute zero is reached after an infinite number of isothermic and adiabatic reversible processes, when the heat theorem is satisfied *S*(0, *x*_1_)=*S*(0, *x*_2_). While on the left, a finite number of steps appears to be sufficient when the heat theorem is violated *S*(0, *x*_1_)>*S*(0, *x*_2_). The problem with this argument is that the last adiabat is impossible, because it must preserve the probability distribution set by the last isotherm, which is not confined to the ground-space. [Figure, courtesy Wikipedia Foundation].

It is worth noting that scaling we find, for example, the inverse polynomial of equation (1), is more benign than what one might have feared, and does not exclude obtaining lower temperatures with a modest increase of resources. However, it is also stronger than that envisioned when the third law was original formulated. Consider for example, the cooling protocol of Fig. 1 proposed by Nernst. It is unphysical, since it requires an infinite number of heat baths, each one at a lower and lower temperature, and with the final heat baths at close to zero temperature. However, it allows the temperature of the system to decrease exponentially in the number of steps, something which we are able to rule out when one doesn't have colder and colder reservoirs.

Finally, let us return to the question of the relationship between the unattainability principle and Nernst's heat theorem. In modern terms, the latter can just be understood as saying that the degeneracy of the ground state *g* cannot be changed. This may be partly a matter of definition: if we can change the Hamiltonian then the degeneracy of the ground state can change, although one may then argue that the system is now in a different phase of matter. Regardless, one can still apply our results to this case. As described in Section A of the Supplementary Discussion, this is accomplished by letting *g* and Δ in al the above results correspond to the final Hamiltonian, while the other parameters (*J* and *Z*_S_) correspond to the initial Hamiltonian *H*_S_. Thus, if the heat theorem is violated and we can change *g*, this at best allows us to cool at a faster rate, rather than violate the unattainability principle.

## Methods

### Sketch of the proof

We qualitatively describe the proof technique with a simple case. Consider the transformation of a qubit, from a maximally mixed state to a pure state:


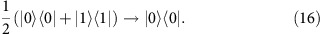


**Figure 2:**
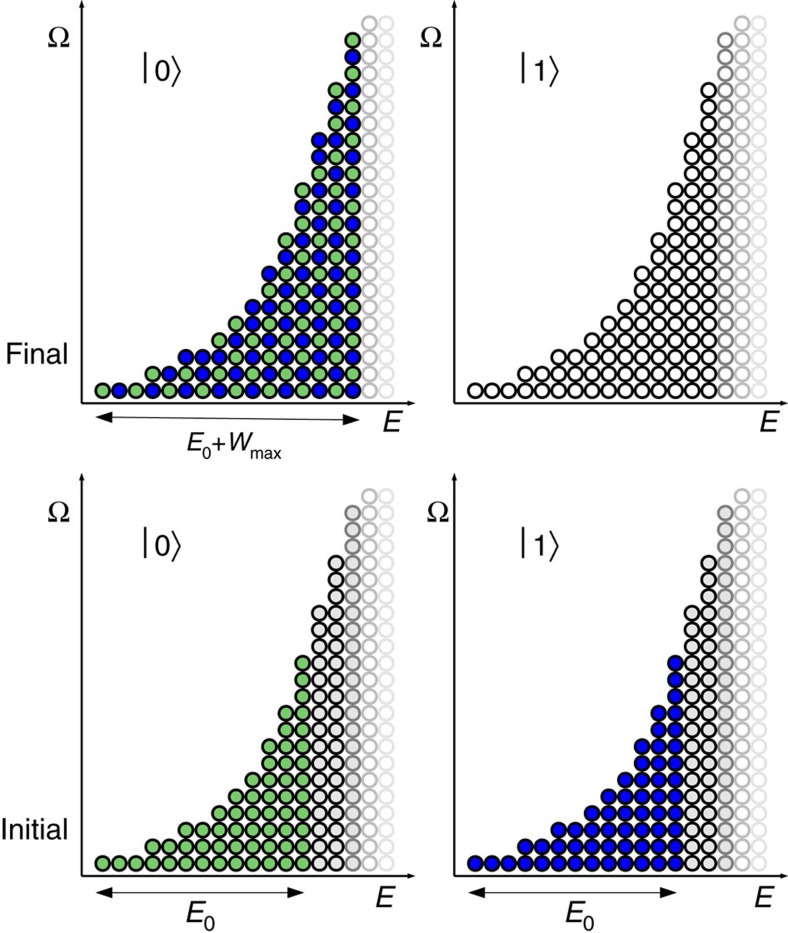
Erasing a qubit. Here we illustrate the limitations of transforming a qubit with *H*_S_=0, from a maximally mixed state to a pure state. Each of the four panels depicts the function Ω(*E*), and each little circle represents a microstate of the bath, having the energy of the corresponding column. The two lower panels together contain all the joint states of system and bath before the transformation: the left(right) panel contains all the states of the bath together with the system being in state |0〉(|1〉). In the same way, the two upper panels contain all the joint states of system and bath after the transformation. The goal of erasure is to put all the states of the two lower panels to the upper-left panel, with the constraint that any state can only be shifted to the right by no more than *w*_max_. Energy *E*_0_, the solution of equation (17), is the threshold below which all states from the lower two panels can be mapped to the upper-left panel. Above *E*_0_, some states will have to be mapped to the upper-right panel, contributing to a non-zero 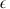.

For simplicity we consider *H*_S_=0, hence this is not really cooling, but rather erasure—however, the essentials are identical. The initial(final) joint microstates of qubit and bath are depicted in the lower(upper) panels of Fig. 2. A perfect implementation of transformation (16) amounts to mapping all the states of the lower panels to the upper-left panel. If the Hilbert space of the bath is finite, such a transformation is incompatible with unitarity, which requires that all final states are occupied. However, an infinite-dimensional bath allows for some final states to not be the image of any initial state. The crucial constraint is that the work that this transformation can consume in the worst case, is bounded by *w*_max_. This restricts the map of every state depicted in the lower panels in Fig. 2, to a state in the upper panels which cannot be shifted to the right by more than *w*_max_. We are interested in the energy *E*_0_, the threshold energy below which all initial states (depicted in the lower two panels) can be mapped to final states (the upper-left panel). *E*_0_ is the solution of


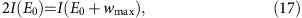


where *I*(*E*) is the number of eigenvalues of *H*_B_ smaller than *E*. The factor of two in equation (17) reflects the fact that there are two initial states of the system, being mapped to one final state of the system. The coloured area of the two lower panels represent the states counted by the left-hand side of equation (17), while the coloured area of the upper-left panel represents the states counted by the right-hand side of equation (17). Above *E*_0_, some states will have to be mapped to the upper-right panel. The error 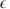 is the sum of the probabilities of all states mapped to the upper-right panel. In this simple case, the probability of a state from the lower panels with energy *E* is 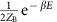 (the factor 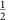 comes from the qubit), which decreases exponentially as we move to the right. The optimal protocol is that which minimizes 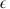, and must satisfy bound (11) (Supplementary Methods). This picture makes clear that the faster Ω(*E*) grows, the larger *E*_0_ is, and the smaller 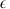 is. But when Ω(*E*) grows exponentially, we have *I*(*E*)∝e^*αE*^, and equation (17) becomes 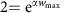, which holds for a finite *w*_max_ independently of *E*_0_. This implies that all states can be mapped to the upper-left, and consequently 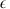
=0. In this and also the super-exponential case, there is no third law. However, such a density of states does not allow for having a well-defined thermal state and partition function at all temperatures. In more physical terms, such a substance would suck up all the energy from the surrounding systems (for certain initial conditions), and also, it would violate Beckenstein's entropy bound^41^. A possible concern of the reader may be that Ω(*E*) is often approximated by an exponential, but this is because the reservoir is often assumed to have infinite volume *V*. Here we argue that in any process implemented in finite time, the system can only interact with a finite region of a bath.

Another way to see the link between *w*_max_ and the achievable temperature 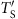 can be seen from the following protocol, recently explored, for example, in ref. 42. Imagine, we have a two level system with energy gap Δ. One cooling method would be to put the system in contact with a bath at temperature *T*, and raise the energy of the excited state by an amount *w*_max_ isothermally. After this, the probability that the system has successfully been put in its ground state is given by 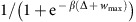. We then remove the system from the heat bath, and then lower the energy of the excited state back to Δ, putting the system into a temperature of 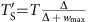. By choosing *w*_max_ large enough, we can make the temperature arbitrarily low, and in this limit, the average work *W* is finite and dominated by the first isothermal step, giving *W*=*T* log(1+e^−*β*Δ^), the change in free energy. Note that the average work *W* is much smaller than the worst-case work *w*_max_. Achieving absolute zero using this protocol clearly requires infinite resources (in this case, raising the excited state an infinite amount). Assumption (13) applied to the above protocol gives us a final temperature


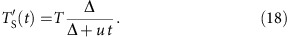


### Data availability

Data sharing not applicable to this article as no datasets were generated or analysed during the current study.

## Additional information

**How to cite this article:** Masanes, L. & Oppenheim, J. A general derivation and quantification of the third law of thermodynamics. *Nat. Commun.* **8,** 14538 doi: 10.1038/ncomms14538 (2017).

**Publisher's note**: Springer Nature remains neutral with regard to jurisdictional claims in published maps and institutional affiliations.

## Supplementary Material

Supplementary Information Supplementary Discussion, Supplementary Methods and Supplementary References.
