# Supplementary material for: A general derivation and quantification of the third law of thermodynamics
Source: Nat Commun. 2017 Mar 14;8:14538. doi: 10.1038/ncomms14538 (PMC5355879; doi:10.1038/ncomms14538)
Supplement: Supplementary Information — Supplementary Discussion, Supplementary Methods and Supplementary References. [file ncomms14538-s1.pdf]

I. SUPPLEMENTARY DISCUSSION

A. The system being cooled

We refer to the system to be cooled as "the system". We assume that the system is finite-dimensional, and treat the infinite-dimensional case in Section I G. We denote its energy eigenstates and eigenvalues by $|s\rangle$ and $\mathcal{E}_s$, where $s = 1, 2, \ldots, d$, such that the Hamiltonian is

$$H_{\mathsf{S}} = \sum_s \mathcal{E}_s |s\rangle\langle s| \ . \tag{1}$$

Without loss of generality $0 = \min_s \mathcal{E}_s$ and define $J = \max_s \mathcal{E}_s$. We denote by g the degeneracy of the ground state, and by Δ the energy gap above it.

The initial state of the system ρ_{S} is arbitrary, with spectral decomposition

$$\rho_{\mathsf{S}} = \sum_s \lambda_s |\phi_s\rangle\langle\phi_s| \ . \tag{2}$$

The results that we obtain depend on the density-matrix's eigenvalues λ_s, but not on its eigenstates $|\phi_s\rangle$. In particular, these can be coherent superpositions of energy eigenstates $|s\rangle$, but this does not affect our bounds.

Special attention is given to the thermal (or Gibbs) state

$$\rho_{\mathsf{S}} = \frac{1}{Z_{\mathsf{S}}} \mathrm{e}^{-\beta_{\mathsf{S}} H_{\mathsf{S}}} \tag{3}$$

at temperature $T_{\mathsf{S}} = 1/\beta_{\mathsf{S}}$. As usual, the normalization factor

$$Z_{\mathsf{S}} = \mathsf{tr}_{\mathsf{S}}\, \mathrm{e}^{-\beta_{\mathsf{S}} H_{\mathsf{S}}} \tag{4}$$

is the partition function. If the final state is thermal, we denote its temperature and partition function by T'_{S} and Z'_{S}.

B. The thermal bath

In order to assist the cooling process there is a bath or reservoir with finite volume V, and hence, finite heat capacity C. The reason for limiting the volume is that, in finite time, the system can only (fully) interact with a bath of finite volume. At the very least, the Lieb-Robinson bound [5] establishes a limit on the speed at which information propagates within a system with local interactions. Roughly, the time it takes for a system to interact with a V-volume bath, in a D-dimensional space, is $t \geq \frac{1}{v} V^{1/D}$, where v is proportional to the "speed of sound" of the bath.

If the volume of the bath is not well defined, then the parameter V can be understood as the number of bosonic or fermionic modes, spins, subsystems, etc. Note that, despite its finite extension, the bath can have an infinite-dimensional Hilbert space. Even more, the bath can be a quantum field, in which case it will have an infinite number of modes. Despite this, the spatial finiteness will warrant that the number of levels with energy below any finite value will remain finite. In the pathological case where there are bosonic modes with zero energy, we ignore them without affecting the cooling properties of the bath. We show below that baths with infinite-dimensional Hilbert space have more cooling capacity.

The energy eigenstates of the bath $|b\rangle$ are labeled by $b = 1, 2, \ldots$, and the corresponding energies by $\mathcal{E}_b$. We assume that $\mathcal{E}_b$ are increasingly ordered, and that $\mathcal{E}_1 = 0$. We assume that the bath is in the thermal or canonical state $\rho_{\mathsf{B}} = \frac{1}{Z_{\mathsf{B}}} \mathrm{e}^{-\beta H_{\mathsf{B}}}$, at temperature $T = \frac{1}{\beta}$, with the normalization factor $Z_{\mathsf{B}} = \mathsf{tr}\, \mathrm{e}^{-\beta H_{\mathsf{B}}}$ being the partition function. The free energy of the canonical state at inverse temperature β is

$$F_{\mathsf{can}} = -\frac{1}{\beta} \ln Z_{\mathsf{B}}(\beta) \ , \tag{5}$$

and the density of free energy is

$$f_{\mathsf{can}} = -\frac{1}{\beta V} \ln Z_{\mathsf{B}}(\beta) \ . \tag{6}$$

The heat capacity of the canonical state is

$$\begin{aligned} C_{\mathsf{can}} &= \frac{\partial \langle E \rangle}{\partial T} \\ &= \sum_b \frac{\mathcal{E}_b\, \mathrm{e}^{-\beta \mathcal{E}_b}}{Z_{\mathsf{B}}} \left[-\mathcal{E}_b + \sum_{b'} \frac{\mathcal{E}_{b'}\, \mathrm{e}^{-\beta \mathcal{E}_{b'}}}{Z_{\mathsf{B}}} \right] \frac{\partial \beta}{\partial T} \\ &= \beta^2 \left(\langle E^2 \rangle - \langle E \rangle^2 \right) \ . \end{aligned} \tag{7}$$

The number of states with energy at most E is denoted by

$$I(E) = |\{b : \mathcal{E}_b \leq E\}| \ . \tag{8}$$

The number of states with energy inside the energy window $(E - \omega, E]$ is denoted by

$$\Omega(E) = I(E) - I(E - \omega) \ , \tag{9}$$

and we refer to it as “the density of states”. As usual, we fix the width of the energy window matching the width of the energy distribution in the canonical state

$$\omega = \sqrt{\langle E^2 \rangle - \langle E \rangle^2} = \frac{\sqrt{C_{\mathsf{can}}}}{\beta} \ . \tag{10}$$

It is often the case that, for large volume V, almost all energy levels in the interval $(E - \omega, E]$ are clustered around the upper limit of the interval E. This is due to the fast increase of the number of levels as the energy grows (see for example [6] for a proof). When this is the case, the quantity $\Omega(E)$ does not depend much on ω.

The logarithm of the density of states is the micro-canonical entropy at energy E

$$S(E) = \ln \Omega(E) \ . \tag{11}$$

It is often the case that, in the large volume limit, micro-canonical and canonical entropies become equal. This is proven in [6] for the case of local Hamiltonians. The function $S(E)$ is discontinuous, but it is usually the case that, for sufficiently large V, the relative size of its discontinuities is very small, and the quotient $\frac{S(E)}{V}$ tends to a smooth function of the energy density E/V (see [6]). However, here we do not make any smoothness assumption, and hence, use the discrete derivatives

$$S'(E) = \frac{S(E) - S(E - \omega)}{\omega} \ , \tag{12}$$

$$S''(E) = \frac{S(E) + S(E - 2\omega) - 2S(E - 2\omega)}{\omega^2} \ . \tag{13}$$

The temperature of the micro-canonical state with energy E is given by

$$T_{\mathsf{mic}}(E) = \frac{1}{S'(E)} \ , \tag{14}$$

and the corresponding heat capacity is

$$C_{\mathsf{mic}}(E) = \frac{1}{T'_{\mathsf{mic}}(E)} = -\frac{S'^2(E)}{S''(E)} \in [0, \infty) \ . \tag{15}$$

All reasonable systems (black holes excluded) have a positive heat capacity, and a negative one would allow to cool to absolute zero any system, violating the third law. The finiteness of the heat capacity is a consequence of the finite volume of the bath.

The density of free energy for the micro-canonical state at inverse temperature β_0 is

$$f_{\mathsf{mic}}(\beta_0) = \frac{1}{V} \left(E_0 - \frac{1}{\beta} S(E_0) \right) \ , \tag{16}$$

where E_0 is the solution of $S'(E_0) = \beta_0$. Note the difference between the background inverse temperature β and the one defining the state β_0.

C. The cooling process

The cooling process consists of a joint transformation of system, bath and weight. We follow [4] in that the work storage device is modeled by a weight with Hamiltonian

$$H_{\mathsf{W}} = \int_{-\infty}^{\infty} w \, |w\rangle\langle w| \, dw \ , \tag{17}$$

where the orthonormal basis $\{|w\rangle : w \in \mathbb{R}\}$ corresponds to the position of the weight. The Hermitian operator Π translates the energy eigenbasis

$$\mathrm{e}^{ix\Pi}|w\rangle_{\mathsf{W}} = |w+x\rangle_{\mathsf{W}} \ , \tag{18}$$

for all $w, x \in \mathbb{R}$.

In recent approaches to nano-thermodynamics [1–3], the work consumed or generated in a process is constrained to have small fluctuations around its mean value. However, here we want to be maximally general, hence we allow the work consumed to fluctuate arbitrarily. Hence, our weight is an ideal source or sink of work, which can be used to simulate any other work storage device appearing in the literature.

As mentioned in the main text, to transform cooling bounds in terms of work to one in terms of time, we can assume that injecting work into the system or the bath requires an amount of time that grows with the amount of work injected. Hence, since we require the transformation to be implemented within a certain given time, we restrict the worst-case work consumed to be at most a given constant w_{max}. We stress that the work average is not restricted, and in particular, it can be larger than the free energy difference of the transformation, which breaks thermodynamical reversibility.

Abstractly, a cooling process is characterized by a completely positive and trace-preserving map

$$\Gamma_{\mathsf{S}}(\rho_{\mathsf{S}}) = \mathsf{tr}_{\mathsf{BW}}\, \Gamma(\rho_{\mathsf{S}} \otimes \rho_{\mathsf{B}} \otimes \rho_{\mathsf{W}}) \ , \tag{19}$$

satisfying the following requirements:

1. **Microscopic reversibility:** there is a unitary operator

$$U : \mathcal{H}_{\mathsf{S}} \otimes \mathcal{H}_{\mathsf{B}} \otimes \mathcal{H}_{\mathsf{W}} \longrightarrow \mathcal{H}_{\mathsf{S}} \otimes \mathcal{H}_{\mathsf{B}} \otimes \mathcal{H}_{\mathsf{W}} \ , \tag{20}$$

jointly acting on the Hilbert spaces of system, bath and weight. That is

$$\Gamma(\rho_{\mathsf{S}} \otimes \rho_{\mathsf{B}} \otimes \rho_{\mathsf{W}}) = U\, (\rho_{\mathsf{S}} \otimes \rho_{\mathsf{B}} \otimes \rho_{\mathsf{W}})\, U^\dagger \ . \tag{21}$$

2. **Conservation of total energy:**

$$\big[U, H_{\mathsf{S}} + H_{\mathsf{B}} + H_{\mathsf{W}}\big] = 0 \ . \tag{22}$$

3. **Independence of the weight's "position":**

$$[U, \Pi] = 0 \ . \tag{23}$$

4. **Bound on worst-case work transferred:**

$$\begin{aligned} &U : \mathcal{H}_{\mathsf{S}} \otimes \mathcal{H}_{\mathsf{B}} \otimes |w\rangle_{\mathsf{W}} \longrightarrow \\ &\mathcal{H}_{\mathsf{S}} \otimes \mathcal{H}_{\mathsf{B}} \otimes \mathsf{span}\big\{|w'\rangle_{\mathsf{W}} : |w'-w| \leq w_{\mathsf{max}}\big\} \end{aligned} \tag{24}$$

for all $w \in \mathbb{R}$.

The first point follows from the fact that closed systems evolve according to the Schrödinger Equation. The second point expresses the conservation of energy (First Law of Thermodynamics). As noted in the main section, this is not a restrictive assumption, and just ensures that we account for all sources of energy.

The third point ensures that the weight is only used as a source of work, and not, for instance, as an entropy dump [4]. More concretely, in Section II A it is proven that, any U satisfying (23) induces a map on system and bath which is a mixture of unitaries:

$$\mathsf{tr}_{\mathsf{W}}\big(U \rho_{\mathsf{S}B} \otimes \rho_{\mathsf{W}} U^\dagger\big) = \int dw\, p(w)\, u_w \rho_{\mathsf{SB}} u_w^\dagger \ . \tag{25}$$

This prevents the entropy of system and bath to decrease (Second Law of Thermodynamics). Essentially, for the work system, all that should matter is how much work is delivered to our system, thus energy differences matter, but the zero of the energy should not.

The energy that is subtracted from or added to the weight is in general not fixed, it fluctuates depending on the micro-state of system and bath. However, we assume that in finite time, the worst-case work fluctuation is bounded by a given value w_{max}. This is encapsulated in point four. We assume that the variable w_{max} can increase with the time invested in the transformation.

D. Summary of the Argument

In this subsection we summarize the derivation of the quantitative third law by presenting the steps of the proof as a series of results. The proofs are given in Section II.

We start by showing that the action of U on system and bath (when the weight is traced out) cannot reduce their entropy. In particular, this prevents to use the weight as an entropy dump.

Result 1 *If* $[U,\Pi]=0$ *then for every state of the weight* ρ_{W} *there is a probability distribution* $p(w)$ *such that*

$$\begin{aligned}\Gamma_{\mathsf{SB}}(\rho_{\mathsf{SB}}) &= \mathrm{tr}_{\mathsf{W}}\big(U\rho_{\mathsf{SB}}\otimes\rho_{\mathsf{W}}U^\dagger\big)\\ &= \int dw\, p(w)\, u_w\rho_{\mathsf{SB}}u_w^\dagger\ ,\end{aligned} \tag{26}$$

where u_w *are unitaries.*

As a result, the action of the cooling process on the system and bath is at best unitary (which will preserver the Von Neumann entropy), and possibly entropy increasing if a mixture of unitaries. As we will show in Section II A, the unitaries u_ω depend on the global unitary U, but not on the state of the weight ρ_{W}.

The error of the cooling process is quantified by the probability of not being in the ground space

$$\epsilon = 1-\mathrm{tr}[P\,\Gamma_{\mathsf{S}}(\rho_{\mathsf{S}})]\ , \tag{27}$$

where P is the projector onto the ground space of H_{S} and Γ_{S} is the action of the cooling process on the system

$$\Gamma_{\mathsf{S}}(\rho_{\mathsf{S}}) = \mathrm{tr}_{\mathsf{BW}}\big(U\rho_{\mathsf{S}}\otimes\rho_{\mathsf{B}}\otimes\rho_{\mathsf{W}}U^\dagger\big)\ . \tag{28}$$

For the following result it is useful to define

$$\xi = J + T\ln\frac{\lambda_{\mathsf{max}}}{\lambda_{\mathsf{min}}} + w_{\mathsf{max}}\ , \tag{29}$$

where $\lambda_{\mathsf{max}}, \lambda_{\mathsf{min}}$ are the largest and smallest eigenvalues of ρ_{S}.

Result 2 *The error made when cooling a system to absolute zero satisfies*

$$\epsilon \geq \frac{\mathrm{e}^{-\beta(E_0+\omega)}}{Z_{\mathsf{B}}}\lambda_{\mathsf{min}}\left[d\Omega(E_0+\omega) - g\Omega(E_0+\xi+\omega)\right] \tag{30}$$

where E_0 *is the smallest energy violating*

$$dI(E) \leq g\, I(E+\xi)\ . \tag{31}$$

Recall that ω is defined in (10). The dependence of (30) and (29) on the smallest eigenvalue λ_{min} makes ϵ a discontinuous function of the state ρ_{S}, which is unphysical. In Section I G we apply a standard smoothing technique to make ϵ a continuous function of the state. This also allows us to adapt our results to infinite-dimensional systems ($d\to\infty$).

The above bound on ϵ is valid with full generality. However, solving equation (31) is in general difficult. Next we assume the positivity of the (micro-canonical) heat capacity and derive a more usable bound.

Result 3 *The error made when cooling a system to absolute zero satisfies*

$$\epsilon \geq \frac{1}{Z_{\mathsf{B}}}\mathrm{e}^{-\beta(E_0+\omega)+S(E_0+\omega)}\,\lambda_{\mathsf{min}}\,\frac{d}{3}\ , \tag{32}$$

where E_0 *is the (unique) solution of*

$$S'(E_0)\,\xi = \ln \frac{2\,d}{3\,g} \ , \tag{33}$$

provided

$$\frac{T}{\sqrt{2}}\, C_{\mathsf{can}}^{1/2}(V) > J + T \ln \frac{\lambda_{\mathsf{max}}}{\lambda_{\mathsf{min}}} + w_{\mathsf{max}} \ , \tag{34}$$

and

$$0 \leq C_{\mathsf{mic}}(E) < \infty \ , \tag{35}$$

for all E.

Our bound (32) depends implicitly on the two parameters V and w_{max}, which quantify the amount of resources. But bound (32) is valid in the range of parameters (V and w_{max}) satisfying condition (34). However, this regime includes the late time situations we are interested in, since we can take the volume of the bath V sufficiently large, and $C_{\mathsf{can}}(V)$ grows with the volume in at least a linear rate.

An important consequence of Result 3 is the following. The faster $\Omega(E)$ or $S(E)$ grow, the slower $S'(E)$ decreases and the larger the solution E_0 of equation (33) is. A large E_0 in (32) gives a smaller bound for ϵ. As we will see in Result 6, baths with faster Ω-growth allow for better cooling. And actually, when this growth is exponential or faster then perfect cooling ($\epsilon = 0$) can be achieved with finite w_{max} (see Section I H). However, these cases correspond to negative heat capacity, which is unphysical.

Result 4 *If the final state is thermal, then its temperature* T'_{S} *satisfies*

$$T'_{\mathsf{S}} \geq \frac{\Delta}{\ln \frac{d}{g\,\epsilon}} \ , \tag{36}$$

where ϵ *is the probability of the system not being in the ground state* (32)*, and* Δ *is the energy of the first excited state above the ground space of the system.*

The following result applies Result 3 to the case where the initial state of the system is thermal at temperature T_{S}, in which case we have

$$\xi = J + \frac{T}{T_{\mathsf{S}}} J + w_{\mathsf{max}} \ . \tag{37}$$

It also uses Result 4 to translate the error probability ϵ to the final temperature of the system T'_{S}.

Result 5 *If the microcanonical entropy of the bath scales as*

$$S(2E, 2V) = 2S(E, V) \tag{38}$$

then, the final temperature of the system cannot be lower than

$$T'_{\mathsf{S}} \ \geq \ \frac{T\Delta}{V\left[f_{\mathsf{mic}}(\frac{1}{\xi}\ln\frac{2\,d}{3\,g}) - f_{\mathsf{can}}(\beta)\right] + \frac{T}{T_{\mathsf{S}}} J + T \ln \frac{3d}{g}} \tag{39}$$

where only leading terms in V *are considered, and we take the regime given by Equations* (34) *and* (35)*.*

This result appears in the main section, as Equation (7). Here we can observe the very natural fact that: the smaller the initial temperature T_{S} is, the smaller the final temperature T'_{S} becomes. The above result is already a third law, in the sense that it places a limitation on the temperature which is achievable, given a restriction on resources V and w_{max}. Also note that, in most known types of bath, when V is large we have $f_{\mathsf{mic}}(\beta_0) \approx f_{\mathsf{can}}(\beta_0)$, and no distinction between the two free energy densities is necessary.

In what follows we consider a family of entropy functions for the bath. In particular, this family contains the entropy of a box of electro-magnetic radiation in D spatial dimensions and volume V (in the large V limit). This result illustrates, that the faster the entropy grows (larger $\nu > 0$), the lower the achievable temperature.

Result 6 *If the entropy function of the bath is*

$$S(E) = \alpha V^{1-\nu} E^{\nu} \ , \tag{40}$$

with $\nu \in [1/2, 1)$*, then equation* (39) *becomes*

$$T'_{\mathsf{S}} \ \geq \ \frac{T\Delta}{V\left[\frac{\alpha}{\ln\frac{2}{3}\frac{d}{g}}\,\xi\right]^{\frac{1}{1-\nu}} + \frac{T}{T_{\mathsf{S}}} J + T\ln\frac{3d}{g}} \ , \tag{41}$$

up to leading terms in V *and* ξ*.*

The largest work fluctuation w_{max} and the volume of the bath V are resources that we associate to the time invested in the cooling process. The larger this quantities are, the lower the final temperature can be. In the following result we express the lowest achievable temperature in terms of time. In order to facilitate a simpler expression above, we have suppressed all constant terms.

Result 7 *If the entropy function is* (40) *and*

$$t \ \geq \ \tfrac{1}{v} V^{1/D} \ ,$$
$$t \ \geq \ \tfrac{1}{u} w_{\mathsf{max}} \ ,$$

then

$$T'_{\mathsf{S}} \geq \frac{T\Delta}{v^D}\left[\frac{\ln\frac{2}{3}\frac{d}{g}}{\alpha\, u}\right]^{D+1} \frac{1}{t^{2D+1}} \ , \tag{42}$$

up to leading terms.

E. Cooling processes with non-constant H_{S}

Our previous results only apply to the case where the Hamiltonian of the system is kept constant during the cooling process. However, our results can be easily adapted to the case where the Hamiltonian changes.

It is straightforward to check that, if the parameters g and Δ appearing in our formulae are those of the final Hamiltonian, then Results 2, 3, 4, 5, 6, 7 become true for processes with non-constant H_{S}.

F. Cooling processes which discard part of the system

A well known example of this type of process is evaporative cooling, in which the cooled final system contains only a fraction of the atoms of the initial system. In general, we write the Hilbert space of the initial system $\mathcal{H}_{\mathsf{S}}$ as the product of the final system $\mathcal{H}'_{\mathsf{S}}$ times the discarded part $\mathcal{H}''_{\mathsf{S}}$, that is $\mathcal{H}_{\mathsf{S}} = \mathcal{H}'_{\mathsf{S}} \otimes \mathcal{H}''_{\mathsf{S}}$. This translates into a relation between the respective dimensions of these Hilbert spaces $d = d'd''$.

Now, we can repeat the argument that led to Results 1-7, but replacing $\mathcal{H}^0_{\mathsf{S}}$ by our new target subspace $\mathcal{H}'^0_{\mathsf{S}} \otimes \mathcal{H}''_{\mathsf{S}}$, where $\mathcal{H}'^0_{\mathsf{S}}$ is the ground space of $\mathcal{H}'_{\mathsf{S}}$. Hence, if P' is the projector onto $\mathcal{H}'^0_{\mathsf{S}}$ then the projector onto the target subspace is $P = P' \otimes \mathbb{1}''_{\mathsf{S}}$ where $\mathbb{1}''_{\mathsf{S}}$ is the identity of $\mathcal{H}''_{\mathsf{S}}$. Also, if g and g' are the ranks of P and P', respectively, then we have the relation $g = g'd''$.

Now we apply our results to subspace P. We just have to keep in mind that $d, g, J, \lambda_{\min}, \lambda_{\max}, T_{\mathsf{S}}$ refer to the initial system (before the partial trace), and Δ, T'_{S} refer to the final system. Also note that the dependence of our bounds on d and g is via d/g, hence there is no difference when using either d/g or d'/g'.

G. Continuity and infinite-dimensional systems

Some of our bounds on the error ϵ and the temperature T'_{S} depend on the lowest eigenvalue λ_{min}, which makes them a discontinuous function of the initial state ρ_{S}. For example, Result 3 provides a lower bound for ϵ which tends to zero as λ_{min} tends to zero. Hence, for very small λ_{min} the result is useless.

One way to fix this problem is by truncating the Hilbert space of the system $\mathcal{H}_\mathsf{S}$ such that the smallest eigenvalues of H_S are eliminated. After truncating $\mathcal{H}_\mathsf{S}$, system's parameters λ'_{min}, J' and d' may take new values. The physical meaning of truncation is that the truncated subspace is assumed to be mapped to the ground space without interfering with the map of the untruncated subspace. Hence, the truncated subspace does not contribute to the error ϵ.

Ideally, one should optimize over all possible truncations, until the lower bounds for ϵ and T'_S are maximal. The truncation always has an optimal non-trivial point, because if one truncates everything except for a 1-dimensional subspace then $\epsilon = 0$, which cannot be maximal. Hence, there is an optimal truncation dimension d' in the interval $1 < d' \leq d$ for which ϵ and T'_S are maximal.

This method can also be used to apply all our results to infinite-dimensional systems. Any finite-dimensional truncation gives finite λ'_{min}, J' and d', which provide a non-trivial bound when substituted in any of our results.

Now, let us apply the truncation method to a harmonic oscillator with energy levels $\mathcal{E}_s = s$ for $s = 1, 2, \ldots$, with initial state being a thermal state at the same temperature as the bath $T_\mathsf{S} = T$. This system suffers from the above mentioned two problems: $\lambda_{\mathsf{min}} = 0$ and $d = \infty$. We solve these problems by truncating out all energy levels $s > d'$, obtaining

$$J' = d' \ , \tag{43}$$

$$\lambda'_{\mathsf{min}} = \frac{\mathrm{e}^{-\beta d'}}{Z_\mathsf{S}} \ , \tag{44}$$

$$\xi' = 2d' + w_{\mathsf{max}} \ . \tag{45}$$

Substituting this in (32) we obtain

$$\epsilon \geq \frac{1}{Z_\mathsf{B} Z_\mathsf{S}} \mathrm{e}^{-\beta(E_0+\omega+d')+S(E_0+\omega)} \frac{d'}{3} \ , \tag{46}$$

$$S'(E_0) = \frac{\ln \frac{2\,d'}{3}}{2d' + w_{\mathsf{max}}} \ . \tag{47}$$

Setting $d' = 2$ gives a non-trivial bound. However, to obtain the optimal value of d', one needs to jointly optimize the above two equations.

H. Negative heat capacity allows perfect cooling

In this subsection we show that when the heat capacity of the bath is negative

$$C_{\mathsf{mic}}(E) < 0 \ , \tag{48}$$

for all E, then perfect cooling to absolute zero ($\epsilon = 0$) with finite w_{max} is possible. Using relation (15) we see that the violation of the above conditions implies $S''(E) \geq 0$. Which in turn implies that $S(E)$ grows at least linearly, or that $\Omega(E)$ grows at least exponentially.

First note that the integral $I(E)$ never grows slower than $\Omega(E)$. Hence $I(E)$ is also exponential or super-exponential. This implies that for any value of d/g there is a sufficiently large energy w_{max} such that

$$\frac{I(E + w_{\mathsf{max}})}{I(E)} \geq \frac{d}{g} \ , \tag{49}$$

for all E. Therefore the smallest energy violating (49) is $E_0 = \infty$, which, when substituted in (30), gives $\epsilon = 0$. In other words, the whole space $\mathcal{H}_\mathsf{S} \otimes \mathcal{H}_\mathsf{B}$ can be mapped into the ground space $\mathcal{H}^0_\mathsf{S} \otimes \mathcal{H}_\mathsf{B}$. There is no unattainability principle.

II. SUPPLEMENTARY METHODS

A. The weight as a work storage system (Result 1)

In this subsection we show that, if the global transformation U commutes with the translations on the weight then the effect of U on system and bath is a mixture of unitaries. And hence, it can never decrease the entropy of system and bath, which amounts to a statement of the second law of thermodynamics.

Let U be a unitary acting on system, bath and weight $\mathcal{H}_{\mathsf{SB}} \otimes \mathcal{H}_{\mathsf{W}}$ which commutes with the translations on the weight $[U, \mathbb{1}_{\mathsf{SB}} \otimes \Pi] = 0$. This implies that we can write it as

$$U = \int dx\, A_x \otimes \mathrm{e}^{ix\Pi} \ , \tag{50}$$

where A_x is a family of operators acting on H_{SB}. By imposing unitarity we obtain

$$\mathbb{1}_{\mathsf{SB}} \otimes \mathbb{1}_{\mathsf{W}} = UU^\dagger = \int dx\, dx'\, A_x A_{x'}^\dagger \otimes \mathrm{e}^{i(x-x')\Pi} \ , \tag{51}$$

which implies

$$\int dx\, A_x A_{x+y}^\dagger = \mathbb{1}_{\mathsf{SB}}\, \delta(y) \ , \tag{52}$$

where $\delta(y)$ is the Dirac delta distribution. Now, let us figure out the structure of the reduced map on system and bath:

$$\begin{aligned} \Gamma(\rho) &= \mathsf{tr}_{\mathsf{W}}\big(U\rho \otimes \sigma U^\dagger\big) \\ &= \int dx\, dx'\, A_x \rho A_{x'}^\dagger\ \mathsf{tr}_{\mathsf{W}}\Big(\mathrm{e}^{i(x-x')\Pi}\sigma\Big) \\ &= \int dx\, dx'\, A_x \rho A_{x'}^\dagger \int dk\, p(k)\, e^{ik(x-x')} \end{aligned} \tag{53}$$

where $p(k) = \langle k|\sigma|k\rangle$ is a probability measure and $|k\rangle$ is the eigenstate $\Pi|k\rangle = k|k\rangle$ for all $k \in \mathbb{R}$. Also, note that, in general, the map Γ depends on the state of the weight σ.

If we define the family of operators

$$u_k = \int dx\, A_x\, e^{ikx} \ , \tag{54}$$

then we can write the reduced map as

$$\Gamma(\rho) = \int dk\, p(k)\, u_k \rho\, u_k^\dagger \ . \tag{55}$$

Now we show that the operators u_k are unitary:

$$\begin{aligned} u_k u_k^\dagger &= \int dx\, dx'\, A_x A_{x'}^\dagger e^{ik(x-x')} \\ &= \int dx\, dy\, A_x A_{x+y}^\dagger e^{-iky} \\ &= \int dy\, \delta(y)\, \mathbb{1}\, e^{-iky} = \mathbb{1} \ , \end{aligned} \tag{56}$$

where we have used (52). In summary, for any initial state of the weight σ, the reduced map (55) is a mixture of unitaries. Interestingly, the set of unitaries u_k is independent of σ, but the probability measure $p(k)$ does depend on σ.

B. Optimal cooling (Result 2)

Here, we formalize the intuition described in Figure 1 for quantifying the probability of all the states which cannot be mapped to the ground space.

We define the subspaces with energy lower than a given value E as

$$\mathcal{H}_{\mathsf{S}}^E = \mathsf{span}\{|s\rangle : \mathcal{E}_s \le E\} \subseteq \mathcal{H}_{\mathsf{S}} \ , \tag{57}$$

$$\mathcal{H}_{\mathsf{B}}^E = \mathsf{span}\{|b\rangle : \mathcal{E}_b \le E\} \subseteq \mathcal{H}_{\mathsf{B}} \ , \tag{58}$$

$$\mathcal{H}_{\mathsf{SB}}^E = \mathsf{span}\{|s\rangle|b\rangle : \mathcal{E}_s + \mathcal{E}_b \le E\} \subseteq \mathcal{H}_{\mathsf{S}} \otimes \mathcal{H}_{\mathsf{B}} \ , \tag{59}$$

and recall that

$$\begin{aligned} \mathsf{dim}\,\mathcal{H}_{\mathsf{S}}^{0} &= g\;, &(60)\\ \mathsf{dim}\,\mathcal{H}_{\mathsf{B}}^{E} &= I(E)\;. &(61)\end{aligned}$$

The optimal cooling unitary u is the one that maps the largest amount of probability from $\rho_{\mathsf{S}}\otimes\rho_{\mathsf{B}}$ to the ground space $\mathcal{H}_{\mathsf{S}}^{0}\otimes\mathcal{H}_{\mathsf{B}}$. Hence, it is useful to denote the subspace corresponding to the largest eigenvalues of $\rho_{\mathsf{S}}\otimes\rho_{\mathsf{B}}$ by

$$\begin{aligned}\mathcal{Q}_X &= \mathsf{span}\left\{|\phi_s\rangle|b\rangle : \lambda_s\, p_b \geq \frac{\mathrm{e}^{-\beta X}}{Z_{\mathsf{B}}}\right\}\\ &= \mathsf{span}\big\{|\phi_s\rangle|b\rangle : \mathcal{E}_b \leq X + T\ln\lambda_s\big\} \qquad (62)\\ &\subseteq \mathcal{H}_{\mathsf{S}}\otimes\mathcal{H}_{\mathsf{B}}\;,\end{aligned}$$

where X is a convenient way to parametrize the probability, and we have used $p_b = \frac{1}{Z_{\mathsf{B}}}\mathrm{e}^{-\beta\mathcal{E}_b}$. The dimension of this "large probability" subspace is

$$\begin{aligned}\mathsf{dim}\,\mathcal{Q}_X &= \sum_s I(X + T\ln\lambda_s)\\ &\geq d\, I(X + T\ln\lambda_{\mathsf{min}})\;, \qquad (63)\end{aligned}$$

where $\lambda_{\mathsf{min}} = \min_s \lambda_s$. Using $J = \max_s \mathcal{E}_s$ and (62) we obtain

$$\mathcal{Q}_X \subseteq \mathcal{H}_{\mathsf{S}B}^{X+J+T\ln\lambda_{\mathsf{max}}}\;, \qquad (64)$$

where $\lambda_{\mathsf{max}} = \max_s \lambda_s$. Assumption "Bound on worst-case work transferred", stated in (24), can be written as

$$u(\mathcal{H}_{\mathsf{SB}}^{E}) \subseteq \mathcal{H}_{\mathsf{SB}}^{E+w_{\mathsf{max}}}\;. \qquad (65)$$

Combining the two above equations we obtain

$$u(\mathcal{Q}_X) \subseteq \mathcal{H}_{\mathsf{SB}}^{X+w_0}\;, \qquad (66)$$

where

$$w_0 = J + T\ln\lambda_{\mathsf{max}} + w_{\mathsf{max}}\;. \qquad (67)$$

Note that, if $\mathcal{Q}_X$ is (fully) mapped into the ground space $\mathcal{H}_{\mathsf{S}}^{0}\otimes\mathcal{H}_{\mathsf{B}}$ then (66) implies

$$u(\mathcal{Q}_X) \subseteq \mathcal{H}_{\mathsf{S}}^{0}\otimes\mathcal{H}_{\mathsf{B}}^{X+w_0}\;, \qquad (68)$$

which in turn implies (substituting (63) and (61))

$$dI(X + T\ln\lambda_{\mathsf{min}}) \leq g\, I(X + w_0)\;. \qquad (69)$$

For the subsequent analysis it is convenient to define the threshold value X_0, which is the infimum of the X's violating (69). With this definition, we write the decomposition

$$\mathcal{H}_{\mathsf{S}}\otimes\mathcal{H}_{\mathsf{B}} = Q_{X_0+\omega}\oplus Q_{X_0+\omega}^{\perp} \qquad (70)$$

in orthogonal subspaces.

In order to obtain an upper bound for the amount of probability that can be mapped from $\rho_{\mathsf{S}}\otimes\rho_{\mathsf{B}}$ to the ground space, we assume that there is no constraint on where $\mathcal{Q}_{X_0+\omega}^{\perp}$ is mapped, and that the only constraint on the image of $\mathcal{Q}_{X_0+\omega}$ is (66). However, the definition of X_0 vie (69) prevents mapping all of $\mathcal{Q}_{X_0+\omega}$ into the ground space. Clearly, the optimum is to map the subspace of $\mathcal{Q}_{X_0+\omega}$ containing the largest eigenvalues of $\rho_{\mathsf{S}}\otimes\rho_{\mathsf{B}}$ to the ground space. The complement of this subspace

$$\mathcal{W} = \mathcal{Q}_{X_0+\omega}\ominus u^{-1}\Big(\mathcal{H}_{\mathsf{S}}^{0}\otimes\mathcal{H}_{\mathsf{B}}^{X_0+w_0+\omega}\Big)\;, \qquad (71)$$

cannot be mapped into the ground space. Also, we know it has dimension

$$\begin{aligned}\mathsf{dim}\mathcal{W} &\geq dI(X_0 + T\ln\lambda_{\mathsf{min}} + \omega) - gI(X_0 + w_0 + \omega)\\ &= d\Omega(X_0 + T\ln\lambda_{\mathsf{min}} + \omega) - g\Omega(X_0 + w_0 + \omega)\\ &\quad + dI(X_0 + T\ln\lambda_{\mathsf{min}}) - gI(X_0 + w_0)\\ &\geq d\Omega(X_0 + T\ln\lambda_{\mathsf{min}} + \omega) - g\Omega(X_0 + w_0 + \omega)\end{aligned}$$

where in the last inequality we have used the definition of X_0 via (69). The subspace $\mathcal{W}$ is mapped in the complement of the ground space, and hence, it contributes to the error ϵ. Our lower-bound for ϵ is obtained by only taking into account this contribution.

Equation (62) tells us that the smallest eigenvalue in $\mathcal{Q}_{X_0+\omega}$, and hence in $\mathcal{W}$, is not smaller than $\frac{1}{Z_{\mathsf{B}}}\mathrm{e}^{-\beta(X_0+\omega)}$. Therefore, we can bound ϵ by the product of this number with the dimension of $\mathcal{W}$

$$\begin{aligned} \epsilon \ &\geq \ \frac{\mathrm{e}^{-\beta(X_0+\omega)}}{Z_{\mathsf{B}}} \\ &\quad \times [d\Omega(X_0 + T\ln\lambda_{\mathsf{min}} + \omega) - g\Omega(X_0 + w_0 + \omega)] \\ &= \ \frac{\mathrm{e}^{-\beta(E_0+\omega)}}{Z_{\mathsf{B}}}\lambda_{\mathsf{min}}\,[d\Omega(E_0+\omega) - g\Omega(E_0+\xi+\omega)] \end{aligned} \tag{72}$$

where we have used definitions

$$E_0 \ = \ X_0 + T\ln\lambda_{\mathsf{min}} \ , \tag{73}$$

$$\xi \ = \ J + T\ln\frac{\lambda_{\mathsf{max}}}{\lambda_{\mathsf{min}}} + w_{\mathsf{max}} \ . \tag{74}$$

In these new variables, $E = E_0$ is the smallest energy violating

$$dI(E) \leq gI(E+\xi) \ . \tag{75}$$

C. Simpler bound for the error (Result 3)

In this subsection we derive an upper bound

$$E_1 \geq E_0 \ , \tag{76}$$

which is easier to obtain than solving (75). This bound E_1 is used to write a lower bound for ϵ that is simpler than (72). We start by assuming that E_1 satisfies

$$S'(E_1) > \frac{1.3\,\beta}{\sqrt{C_{\mathsf{can}}}} \ , \tag{77}$$

and later we prove that this assumption reduces to premise (34). On the other hand, premise (35) warrants that

$$S''(E) \leq 0 \ , \tag{78}$$

which it is used below.

Taylor's theorem implies that for any pair $E, \xi > 0$ there is $\xi^* \in [0,\xi]$ such that

$$S(E+\xi) = S(E) + S'(E)\xi + S''(E+\xi^*)\frac{\xi^2}{2} \ . \tag{79}$$

This and (78) imply

$$S(E+\xi) \ \leq \ S(E) + S'(E)\xi \ . \tag{80}$$

Assumption (77) implies $S'(E) > 0$, which together with (80) gives the upper bound

$$\begin{aligned} I(E+\xi) \ &= \ \sum_{k=0}^{\infty}\Omega(E+\xi-\omega k) \\ &\leq \ \sum_{k=0}^{\infty}\mathrm{e}^{S(E)+S'(E)(\xi-\omega k)} \\ &= \ \mathrm{e}^{S(E)+S'(E)\xi}\sum_{k=0}^{\infty}\mathrm{e}^{-S'(E)\omega k} \\ &= \ \frac{\mathrm{e}^{S(E)+S'(E)\xi}}{1-\mathrm{e}^{-S'(E)\omega}} \ . \end{aligned} \tag{81}$$

We can also write the lower bound

$$I(E) \geq \Omega(E) = \mathrm{e}^{S(E)} \ . \tag{82}$$

Substituting bounds (81) and (82) in (75) we obtain

$$d \leq g\, \frac{\mathrm{e}^{S'(E)\,\xi}}{1-\mathrm{e}^{-S'(E)\,\omega}} \ . \tag{83}$$

Substituting $\omega = \sqrt{C}/\beta$ and using assumption (77) we obtain $1-\mathrm{e}^{-S''(E)\sqrt{C}/\beta} > \frac{2}{3}$, which allows us to write (83) as

$$S'(E)\,\xi \geq \ln\frac{2\,d}{3\,g} \ . \tag{84}$$

We define E_1 to be the infimum value of E satisfying

$$S'(E_1)\,\xi = \ln\frac{2\,d}{3\,g} \ . \tag{85}$$

If $S'(E)$ is strictly monotonic, then equation (85) has a unique solution E_1. Let us show that $S'(E)$ is strictly monotonic.

Equation (85) implies $S'(E_1) > 0$, which together with the finiteness of the micro-canonical heat capacity (35) forces $S''(E_1) < 0$. And this in turn implies the strict decreasing monotonicity of $S'(E)$ around E_1.

Substituting (85) in (77) gives $\sqrt{C} > 1.3\,\beta\,\xi$, which is premise (34). This proves our previous claim: assumption (77) reduces to premise (34).

Now, substituting E_1 in (72) and using (80) we obtain

$$\begin{aligned} \epsilon \ &\geq \ \frac{\mathrm{e}^{-\beta(E_1+\omega)}}{Z_{\mathsf{B}}}\lambda_{\mathsf{min}}\left[d\,\mathrm{e}^{S(E_1+\omega)} - g\,\mathrm{e}^{S(E_1+\xi+\omega)}\right] \\ &\geq \ \frac{\mathrm{e}^{-\beta(E_1+\omega)}}{Z_{\mathsf{B}}}\lambda_{\mathsf{min}}\,\mathrm{e}^{S(E_1+\omega)}\left[d - g\,\mathrm{e}^{S'(E_1+\omega)\xi}\right] \ , \end{aligned}$$

and using the monotonicity of $S'(E)$ we get

$$\begin{aligned} \epsilon \ &\geq \ \frac{\mathrm{e}^{-\beta(E_1+\omega)}}{Z_{\mathsf{B}}}\lambda_{\mathsf{min}}\,\mathrm{e}^{S(E_1+\omega)}\left[d - g\,\mathrm{e}^{S'(E_1)\xi}\right] \\ &= \ \frac{1}{Z_{\mathsf{B}}}\mathrm{e}^{-\beta(E_1+\omega)+S(E_1+\omega)}\,\lambda_{\mathsf{min}}\,\frac{d}{3} \ , \end{aligned} \tag{86}$$

where the equality follows from (85).

D. Relationship between error and temperature (Result 4)

In this subsection we assume that the final state is thermal at temperature T'_{S} and has partition function $Z'_{\mathsf{S}} = Z_{\mathsf{S}}(T'_{\mathsf{S}})$. Because of our convention $\min_s \mathcal{E}_s = 0$, we have

$$\epsilon = 1 - \frac{g}{Z'_{\mathsf{S}}} \ . \tag{87}$$

In principle, the function $Z_{\mathsf{S}}(T'_{\mathsf{S}})$ can be inverted, and the bound for ϵ (32) can be transformed into a bound for T'_{S}. However, this is in general a hard task. In what follows we obtain a general relation between Z'_{S} and T'_{S} which avoids having to invert $Z_{\mathsf{S}}(T'_{\mathsf{S}})$.

For any Hamiltonian H_{S} following convention $\min_s \mathcal{E}_s = 0$ we have

$$Z'_{\mathsf{S}} \leq g + d\mathrm{e}^{-\Delta/T'_{\mathsf{S}}} \ . \tag{88}$$

Combining this with (87) we obtain

$$\epsilon \leq \frac{d}{g}\mathrm{e}^{-\Delta/T'_{\mathsf{S}}} \ , \tag{89}$$

or equivalently,

$$T'_{\mathsf{S}} \geq \frac{\Delta}{\ln\frac{d}{\epsilon\, g}} \ . \tag{90}$$

E. Cooling the system from T_{S} to T'_{S}

In this section we assume that the initial state of the system is thermal $\lambda_s = \frac{1}{Z_{\mathsf{S}}} \mathrm{e}^{-\mathcal{E}_s/T_{\mathsf{S}}}$ at temperature T_{S}. In this case we have

$$\lambda_{\mathsf{max}} = \frac{1}{Z_{\mathsf{S}}} , \tag{91}$$

$$\lambda_{\mathsf{min}} = \frac{\mathrm{e}^{-J/T_{\mathsf{S}}}}{Z_{\mathsf{S}}} , \tag{92}$$

$$\xi = J + \frac{T}{T_{\mathsf{S}}} J + w_{\mathsf{max}} . \tag{93}$$

Substituting these in (86) and (90), we obtain

$$\begin{aligned} T'_{\mathsf{S}} &\geq \frac{\Delta}{\beta(E_1+\omega) - S(E_1+\omega) + V\beta|f_{\mathsf{can}}| + \frac{1}{T_{\mathsf{S}}} J + \ln \frac{3Z_{\mathsf{S}}}{g}} \\ &\geq \frac{T\Delta}{E_1 + \omega - TS(E_1+\omega) + V|f_{\mathsf{can}}| + \frac{T}{T_{\mathsf{S}}} J + T \ln \frac{3d}{g}} \end{aligned} \tag{94}$$

where we have used $Z_{\mathsf{S}} \leq d$ and (6).

F. Non-decreasing $S(E)$ case

In this section we consider the case where the density of states $\Omega(E)$ is non-decreasing for all the energy range of the bath. This is equivalent to not having negative temperature, or $S'(E) \geq 0$. This usually happens in many-body systems with an infinite Hilbert space dimension, like coupled oscillators, electro-magnetic radiation, or any quantum field theory containing bosons. Clearly, most thermal baths are of this sort.

As far as we know, a many-body system has non-decreasing $S(E)$ if an only if its constituents have infinite-dimensional Hilbert space (e.g. bosons). Hence, the content of this subsection does not hold if the constituents of the bath are only spins or fermions. However, for this last case, one can simplify Result 3 by noting that the average energy corresponding to the infinite temperature state constitutes an upper bound for E_0, irrespectively of ξ.

In the case $S'(E) > 0$ we have

$$S(E_1 + \omega) \geq S(E_1) . \tag{95}$$

Using this and the value of ω set in (10) in (86) gives

$$\epsilon \geq \frac{1}{Z_{\mathsf{B}}} \mathrm{e}^{-\beta E_1 + S(E_1) - \sqrt{C_{\mathsf{can}}}} \lambda_{\mathsf{min}} \frac{d}{3} . \tag{96}$$

Using the expressions for the canonical (6) and micro-canonical (16) densities of free energy we can write the above as

$$\epsilon \geq \mathrm{e}^{-\beta V \left[f_{\mathsf{mic}}\left(\frac{1}{\xi} \ln \frac{2}{3}\frac{d}{g}\right) - f_{\mathsf{can}}(\beta)\right] - \sqrt{C_{\mathsf{can}}}} \lambda_{\mathsf{min}} \frac{d}{3} , \tag{97}$$

where we have used that the micro-canonical inverse temperature corresponding to E_1 satisfies (85).

All this procedure can also be applied to (94) instead of (86), giving

$$T'_{\mathsf{S}} \geq \frac{T\Delta}{V\left[f_{\mathsf{mic}}\left(\frac{1}{\xi} \ln \frac{2}{3}\frac{d}{g}\right) - f_{\mathsf{can}}(\beta)\right] + \sqrt{C_{\mathsf{can}}} + \frac{T\,J}{T_{\mathsf{S}}} + T \ln \frac{3d}{g}} \tag{98}$$

It is usually the case that C_{can} grows with the volume slower than V^2. Hence, keeping only leading terms we obtain

$$T'_{\mathsf{S}} \geq \frac{T\Delta}{V\left[f_{\mathsf{mic}}\left(\frac{1}{\xi} \ln \frac{2}{3}\frac{d}{g}\right) - f_{\mathsf{can}}(\beta)\right] + \frac{T}{T_{\mathsf{S}}} J + T \ln \frac{3d}{g}} \tag{99}$$

G. Extensive bath (Result 5)

The above formula can also be achieved by assuming

$$S(2E, 2V) = 2S(E, V) \tag{100}$$

instead of $S'(E) \geq 0$. The scaling law (100) implies

$$S(E, V) = V s(E/V) \ , \tag{101}$$

where $s(E/V)$ is the entropy density. Hence, the solution E_1 of (85) satisfies

$$s'(E_1/V) = \ln \frac{2\,d}{3\,g} \ , \tag{102}$$

which implies $E_1 \propto V$. Relation (100) also implies $C_{\mathsf{can}} \propto V$ and $\omega \propto \sqrt{V} \ll V$. Hence, inequality (95) is satisfied because $E_1 \propto V$ and

$$\begin{aligned} S(E_1 + \omega) &= V s\Big(E_1/V + \mathcal{O}(V^{-1/2})\Big) \\ &\approx V s(E_1/V) \\ &= S(E_1) \ . \end{aligned} \tag{103}$$

Therefore, with assumption (100) instead of $S'(E) \geq 0$, we can also arrive at (99).

H. Radiation-type bath (Result 6)

In this section we consider a family of bath systems in which the micro-canonical entropy has the form

$$S(E) = \alpha\, V \left(\frac{E}{V}\right)^{\nu} \ , \tag{104}$$

where $\alpha > 0$ and $\nu \in [1/2, 1)$ are two constants. A physical system obeying this is electro-magnetic radiation in the large volume limit. In a box of volume V and D spatial dimensions the value of ν for the electro-magnetic radiation is $\nu = \frac{D}{D+1}$. This is thought to be the most entropic bath for its energy and volume[7], and one therefore expects that it is the most advantageous for cooling.

This entropy function has micro-canonical inverse temperature

$$S'(E) = \alpha\, \nu \left(\frac{E}{V}\right)^{\nu-1} \ , \tag{105}$$

which is always positive (the premise of Result 5). The corresponding heat capacity is

$$C_{\mathsf{mic}}(E) = \frac{\alpha\, \nu}{1-\nu}\, V \left(\frac{E}{V}\right)^{\nu} \tag{106}$$

where we have used formula (15). Note that this heat capacity is positive, finite and proportional to the volume.

The solution of equation

$$S'(E_0) = \frac{1}{\xi} \ln \frac{2\,d}{3\,g} \tag{107}$$

is

$$E_0 = V \left(\frac{1}{\alpha\, \nu\, \xi} \ln \frac{2\,d}{3\,g}\right)^{\frac{1}{\nu-1}} \ , \tag{108}$$

and gives

$$S(E_0) = \alpha V \left(\frac{1}{\alpha\, \nu\, \xi} \ln \frac{2\,d}{3\,g}\right)^{\frac{\nu}{\nu-1}} \ . \tag{109}$$

Substituting in the formula for the micro-canonic free energy density (16), and keeping only leading terms in ξ, we obtain

$$f_{\mathsf{mic}}(\tfrac{1}{\xi}\ln\tfrac{2\,d}{3\,g}) \;=\; \left(\frac{\alpha\,\nu\,\xi}{\ln\frac{2\,d}{3\,g}}\right)^{\frac{1}{1-\nu}} - \frac{\alpha}{\beta}\left(\frac{\alpha\,\nu\,\xi}{\ln\frac{2\,d}{3\,g}}\right)^{\frac{\nu}{1-\nu}}$$
$$\approx \left(\frac{\alpha\,\nu\,\xi}{\ln\frac{2\,d}{3\,g}}\right)^{\frac{1}{1-\nu}} \;. \tag{110}$$

The canonical free energy density (6) for the case (104) is

$$f_{\mathsf{can}}(\beta) = -\frac{1}{\beta V}\ln\int dx\; \mathrm{e}^{\beta V\left[\frac{\alpha}{\beta}x^{\nu}-x\right]} \;, \tag{111}$$

where $x = E/V$. In the large V limit we can use the saddle point method, obtaining

$$f_{\mathsf{can}}(\beta) = \left(\frac{\alpha}{\beta}\right)^{\frac{1}{1-\nu}}\left[\nu^{\frac{1}{1-\nu}} - \nu^{\frac{\nu}{1-\nu}}\right] \;, \tag{112}$$

which is just a constant. Therefore, substituting in (99) and considering the large ξ limit, we obtain

$$T'_{\mathsf{S}} \;\geq\; \frac{T\Delta}{V\left[\frac{\alpha\,\nu}{\ln\frac{2\,d}{3\,g}}\,\xi\right]^{\frac{1}{1-\nu}} + \frac{T}{T_{\mathsf{S}}}J + T\ln\frac{3d}{g}}$$
$$\geq\; \frac{T\Delta}{V\left[\frac{\alpha}{\ln\frac{2\,d}{3\,g}}\,\xi\right]^{\frac{1}{1-\nu}} + \frac{T}{T_{\mathsf{S}}}J + T\ln\frac{3d}{g}} \;, \tag{113}$$

where in the last inequality we have used $\nu \leq 1$.

I. Explicit dependence on time (Result 7)

Now, let us assume that the bath is a D-dimensional box of volume V containing radiation at temperature T. In addition we assume

$$t \;\geq\; \tfrac{1}{v}\,V^{1/D} \;, \tag{114}$$
$$t \;\geq\; \tfrac{1}{u}\,w_{\mathsf{max}} \;, \tag{115}$$

and substitute in (113), obtaining

$$T'_{\mathsf{S}} \;\geq\; \frac{T\Delta}{(vt)^{D}\left[\frac{\alpha}{\ln\frac{2\,d}{3\,g}}\left(J + \frac{T\,J}{T_{\mathsf{S}}} + ut\right)\right]^{D+1} + \frac{T\,J}{T_{\mathsf{S}}} + T\ln\frac{3d}{g}}$$
$$\approx\; \frac{T\Delta}{v^{D}}\left[\frac{\ln\frac{2\,d}{3\,g}}{\alpha\,u}\right]^{D+1}\frac{1}{t^{2D+1}} \;, \tag{116}$$

where in the last line we only kept the leading terms in t.

III. SUPPLEMENTARY REFERENCES

[1] O. C. O. Dahlsten, R. Renner, E. Rieper, and V. Vedral, New Journal of Physics **13** (May 2011)
[2] M. Horodecki and J. Oppenheim, Nat. Comm. **4**, 2059 (2013).
[3] J. Åberg, Nat. Comm. **4**, 1925 (2013)
[4] P. Skrzypczyk, A. J. Short, and S. Popescu (2013), arxiv:1302.2811
[5] E. H. Lieb and D. W. Robinson, Commun. Math. Phys. **28**, 251257 (1972)
[6] M.P. Müller, E. Adlam, L. Masanes, and N. Wiebe, Comm. Math. Phys. **340**, 2, 499-561 (2015).
[7] W.G. Unruh, and R. Wald, Phys. Rev. D **4**, 942 (1982).
